# Real-time precise microfluidic droplets label-sequencing combined in a velocity detection sensor

**DOI:** 10.1038/s41598-021-97392-3

**Published:** 2021-09-09

**Authors:** R. Zamboni, A. Zaltron, M. Chauvet, C. Sada

**Affiliations:** 1 Physics and Astronomy Department, University of Padova, Via Marzolo 8, 35131 Padova, Italy; 2 Institute of Applied Physics, University of Münster, Corrensstrasse 2/4, 48149 Münster, Germany; 3 FEMTO-ST Institute, UMR 6174, University of Bourgogne Franche-Comté, 15B Avenue des Montboucons, 25000 Besançon, France

**Keywords:** Optofluidics, Applied physics, Characterization and analytical techniques, Integrated optics, Optical sensors

## Abstract

Droplets microfluidics is broadening the range of Lab on a Chip solutions that, however, still suffer from the lack of an adequate level of integration of optical detection and sensors. In fact, droplets are currently monitored by imaging techniques, mostly limited by a time-consuming data post-processing and big data storage. This work aims to overcome this weakness, presenting a fully integrated opto-microfluidic platform able to detect, label and characterize droplets without the need for imaging techniques. It consists of optical waveguides arranged in a Mach Zehnder’s configuration and a microfluidic circuit both coupled in the same substrate. As a proof of concept, the work demonstrates the performances of this opto-microfluidic platform in performing a complete and simultaneous sequence labelling and identification of each single droplet, in terms of its optical properties, as well as velocity and lengths. Since the sensor is realized in lithium niobate crystals, which is also highly resistant to chemical attack and biocompatible, the future addition of multifunctional stages into the same substrate can be easily envisioned, extending the range of applicability of the final device.

## Introduction

Droplets-based microfluidics enabled the scaling of a large variety of biological and chemical protocols into lab-on-a-chip devices^1,2^. In fact, droplets act as confined microreactor for chemical compounds^3^, as well as container for bioparticle^4–6^. For instance, droplets have been exploited as carrier for many biological reactions and operations, such as PCR and DNA analysis^7^, as well as for chemical synthetization processes^8^. In these applications, a real time system able to keep track of the properties of the droplet is essential. In fact, the volume of droplets during such protocols determines the amount of product of a reaction, as well as the precise quantity of reagents. Similarly, the detection of the velocity is crucial for the knowledge of the kinetics of a reaction. Moreover, the high throughput and parallelization of these devices requires a stable and reliable system to label all the units and keep track of the protocols. The detection of these properties is also essential to guarantee a screening on the droplets generated by standard droplet generators, which guarantee a precision about 1% on droplet volume^9^. This value can be improved by screening droplet with a sorting system coupled with reliable detection methods.

Therefore, a sensing system is usually exploited to label and keep track of the morphological droplet properties, that can influence the protocol or the application. The most common systems for the tracking and labelling purposes involve microscopy and fast cameras for the recording of several frames of the same droplet, and post-processing tools for the imaging analysis. Although this standard method is popular due its good performances and the commercial availability of the required instruments, the post-processing times and the bulkiness of the setup are hindering the strengths of the lab-on-a-chip, their portability, as well as the plug&play structure. In order to improve the imaging method, wide range of techniques^10,11^ and software solutions^12,13^ have been employed to achieve a reliable real-time data processing. However, a mandatory steps towards a real portability of the final device is represented by the integration of the detection systems into the chip, by replacing the use of the microscopy setups.

In order to detect the droplets passage, three main different methods have been proposed either based on optical detection or electrical, or on thermal response. The latter detects the thermal variation caused by a droplet flowing inside a microchannel^14^. Electrical-based methods rely on the contrast between the two phases in the electrical properties, measuring the impedance^15,16^ or capacitance^17–20^ or current^21^ or exploiting microwave-based sensing^22^. However, these solutions require calibrations and are limited by the electrical properties of liquids. The most successful approach consists in the optical sensing^23–29^, which are often based on two measurements carried out in different positions of the channel, where the droplets flows^24,25^. These systems currently exploit optical fibers, which led to a step towards the integration and portability of lab-on-a-chips. Nevertheless, the reproducibility ensured by the fabrication is still lacking of stable protocol for the realization of device with reliable alignment and positioning of optical fiber. Fibers’ integration, in fact, requires sophisticated designing and realization of V-grooves holders and time-consuming procedures of alignment and gluing of the fibers in the grooves, resulting in poor reproducibility. This step has been recently overcome by the development of integrated optical waveguides, that ensure to transport and collect light across the channel with high reproducibility of the alignment^30–37^.

**Figure 1:**
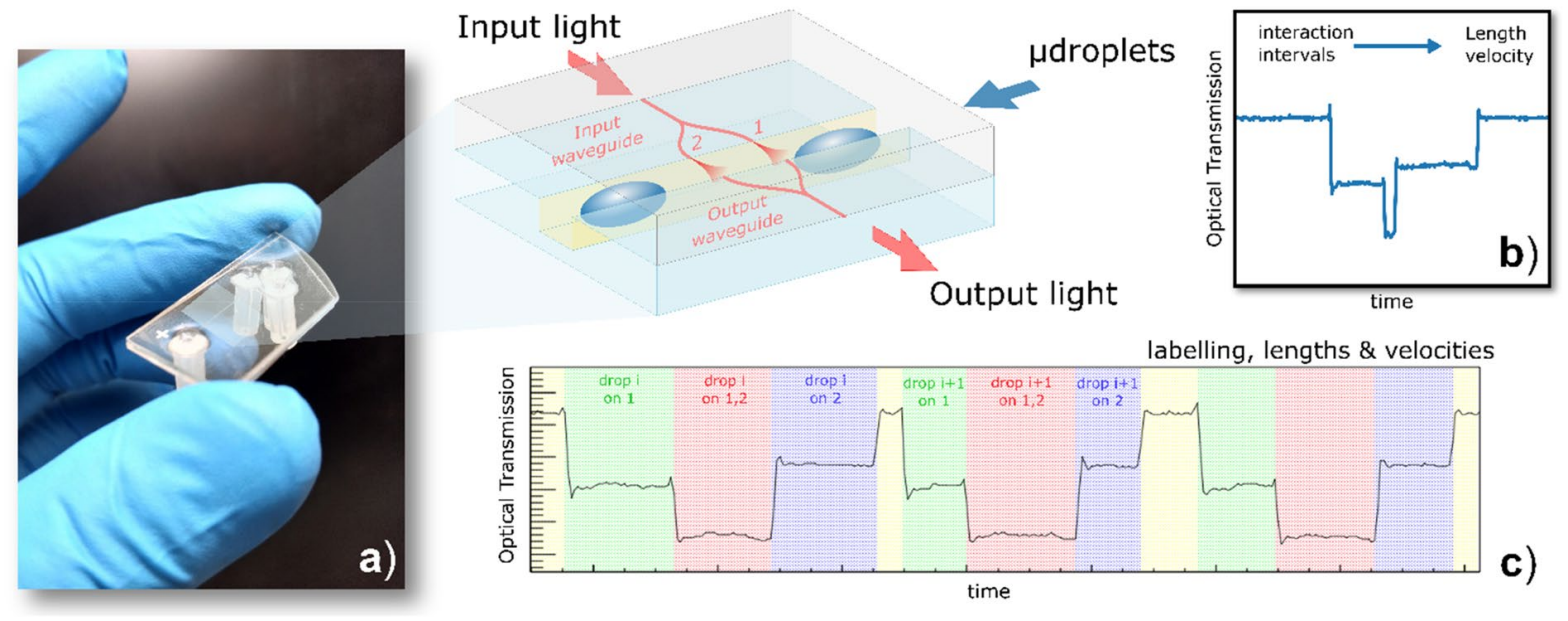
Overview of the MZI integrated system. The picture in (**a**) is the final device with microfluidic circuit and MZI configuration, the sketch evidences the detection of MZI, which splits the light into two arms, both interacting separately with the microfluidic channel and droplets flowing inside. (**b**) and (**c**) report examples of the intensity signal collected at the output of the waveguide when droplets flow inside the microfluidic channel and interacts with light from branches 1 and 2.

In this work, we present an optical integrated approach for achieving a sequential droplets’ labelling identification as well as droplets morphology and dynamics recognition that combines integrated optical waveguides in a Mach–Zehnder interferometer (MZI) configuration in a microfluidic circuitry (Fig. 1a). We integrated MZI configuration consisting of an input waveguide, split in two parallel arms (50–50 MZI-branches^38^) for a certain extent and then, recombined in an output waveguide. The light transmitted by each arm undergoes a different optical path which can be tuned on demand, in order to provide the desired phase and/or intensity modulation when they recombine at the output port. The two branches are crossed by a micro-fluidic channel so that in each branch the light path is interspersed by the droplets flowing within the fluidic channel (Fig. 1b). The light transmitted across the microfluidic channel is therefore collected at the other side and recombined in the output waveguide: many different optical phenomena, such as scattering or absorbance, occurring in the droplets are therefore fully detected. The optical transmission (OT) signal from the MZI, as showed in Fig. 1c, provides trigger-signals each corresponding at each droplet passage. Thanks to the MZI configuration, these trigger-signals provide for further information than the only passage detection as currently done by the approaches already proposed in the literature^27^. As a matter of fact, the OT signal to a sharp variation when the droplets start crossing and pass over the first or second arm of the MZI. These fingerprints enable the measurements of the time intervals between each droplet’s passage allowing the droplet labelling. Moreover, the length and velocity of the droplets can be therefore directly measured without any need of further calibration and signal stabilization. The droplets size dispersion can be quantitatively estimated and the rejection processes of droplets which do not complain with required properties can be easily automatized in a real-time feedback system.

## Materials and methods

### Lithium niobate as substrate

The opto-microfluidic device (Fig. 1a) here proposed has been realized in a monolithic substrate of lithium niobate (LN), a material whose properties go well beyond microfluidics applications and ensure the integration of several functionalities. In fact, Lithium Niobate is a well-known material for the creation of stages for integrated optics^39,40^ and, more recently, for actuating micro- and nano-objects, thanks to its physical properties such as pyroelectricity^41–43^, piezoelectricity^44,45^ and photorefractivity^46, 47^. Furthermore, the feasibility to integrate such optical and manipulation stages in a fluidic circuit^34,48–50^ has definitively contributed to include LN among the materials eligible for realizing lab-on-a-chips demonstrating to be more versatile and flexible in multi-functional platforms’ delivery respect to any material in which optical waveguide^30–32,37^ have been already employed. Specifically, in this work we realized the MZI configuration by means of titanium in-diffusion, which is a standard technique for the realization of waveguides in this material^39^. The device is integrated with a microfluidic circuit which consists in a cross-junction droplet generator and a straight channel.

### Design and fabrication of the device

The Mach Zehnder interferometer has been realized by means of thermal diffusion of 5 µm wide titanium stripes inside a sample of 20 × 30 cm^2^ size lithium niobate sample obtained by commercial x-cut wafer (Crystal Technology Inc.), as described in our previous paper^27^. In this configuration, z-propagating Ti-diffused single mode at 633 nm waveguides in lithium niobate have been realized with an effective numerical aperture NA_eff_ = 0.13^51^. The distance between the two arms (2 W) has been set at (42 ± 1) µm to prevent a cross-coupling of the light exiting the two input arms and coupling into the output waveguides, due to a wider light cone’s aperture respect to the arms distances (further details on the Mach Zehnder design and fabrication process can be found in Supplementary Information). The lithium niobate samples with the integrated MZI waveguides are engraved with a cross shape 200 × 100 µm^2^ channel structure by means of a precision saw equipped with a polishing blade (DISCO DAD 3350); the wall roughness of final microfluidic channel is reported in our previous work^49^. The final in depth profile of Ti concentration in the waveguides has been measured by Secondary Ion Mass Spectrometry (SIMS), and estimated to extend by 2.0 ± 0.2 µm under the diffusion surface, thus facing the top edge of the 100 µm depth channel^24,48^. The sealing with a glass cover is achieved by UV-curable glue bonding^52^. The final structure has been demonstrated to work as droplet generator by our previous work^49^.

### Opto-microfluidic setup

**Figure 2:**
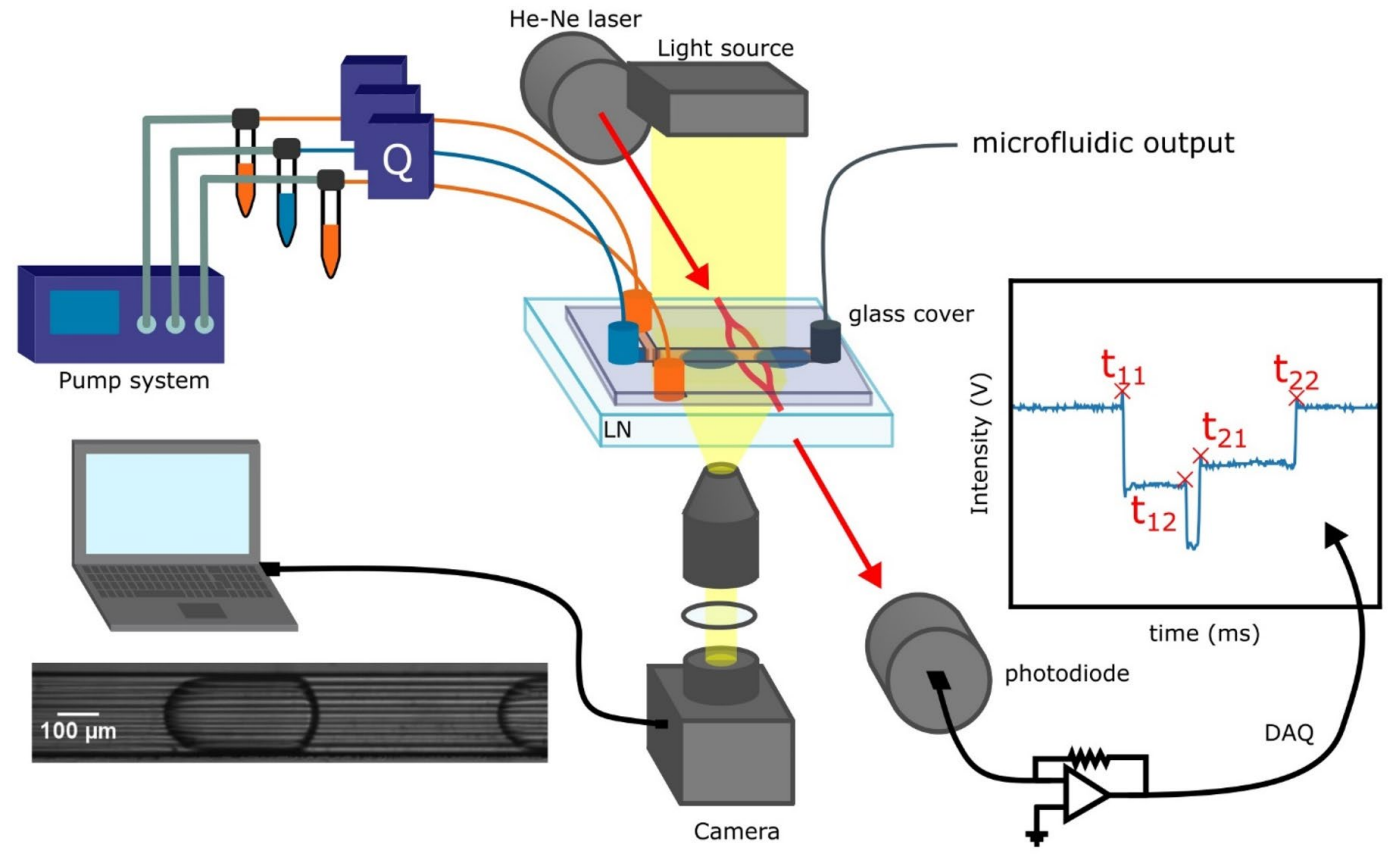
Sketch of optofluidic setup used for the comparison between standard microscope-based imaging system and MZI device. The microfluidic setup consists in a pressure pump equipped with flow sensors to work in feedback. The optical setup is based on the coupling of a pig-tailed He–Ne laser with the MZI, and a photodiode is used to collect the light exiting the waveguide. The current signal from the photodiode is transduced with a transimpedance amplifier and collect using a Data Acquisition System (DAQ). The standard setup used for comparison is represented by a standard microscope setup (LED white source, Objective 10 × /0.25 Nikon with proper tube lens).

The flow rates are controlled by a pressure pump OB1 MK3 (Elveflow, Paris, France) in feedback with flowmeters BFS Coriolis (Bronkhorst, AK Ruurlo, Holland), as depicted in Fig. 2. The optical setup consists of a He–Ne 632.8 nm, 1 mW laser coupled to the input MZI waveguide and collected at the output to a photodiode amplified by a transimpedance and finally digitalized by a NI 6023 D/A converter (200 kHz bandwidth and 0.0023 mV sensibility, NIST). A standard imaging setup video has recorded the droplets displacement inside the channel in a synchronous way in order to compare the performances of the opto-microfluidic device with the imaging approach widely used by the scientific community. The camera used is a fast camera acA800-510um (Basler, Ahrensburg, Germany; 511 fps at maximum resolution 800 × 600) coupled with an objective (10 × /0.25 Nikon) and tube. The video analysis of the droplets is performed by an ad-hoc tracking software as well as the OT signal from the MZI.

The presented device has been tested by droplets produced by Cross-flow junction injecting two immiscible fluids (further details on droplets generation can be found in Supplementary Information). MilliQ® water (refractive index of 1.333 for wavelength of 632.8 nm) has been injected in the central channel of the cross-shape with flowrates varied between [10:55] μL/min. Similarly, Hexadecane oil (Sigma Aldrich, refractive index of 1.434 for wavelength of 632.8 nm) with 3% (w/w) concentration of SPAN80 (Sigma Aldrich) has been flowed in the two orthogonal channel respect to the water channel with a flowrates range of [10:125] μL/min for each of the two channels. The surface tension between water and the hexadecane with that surfactant concentration was measured by pendant drop method and results 4.27 ± 0.04 mN/m.

This configuration allows generating emulsions of similar droplets, exploited for testing the performance of detection of both single droplets and emulsions. The device performance in labelling and detection has been also characterized by sequence of droplets with random properties. The droplets, in this case have been generated by the same configuration applying pressure manually by a syringe filled with air instead of water, in order to obtain a randomness in the bubble properties.

## Working principle

**Figure 3:**
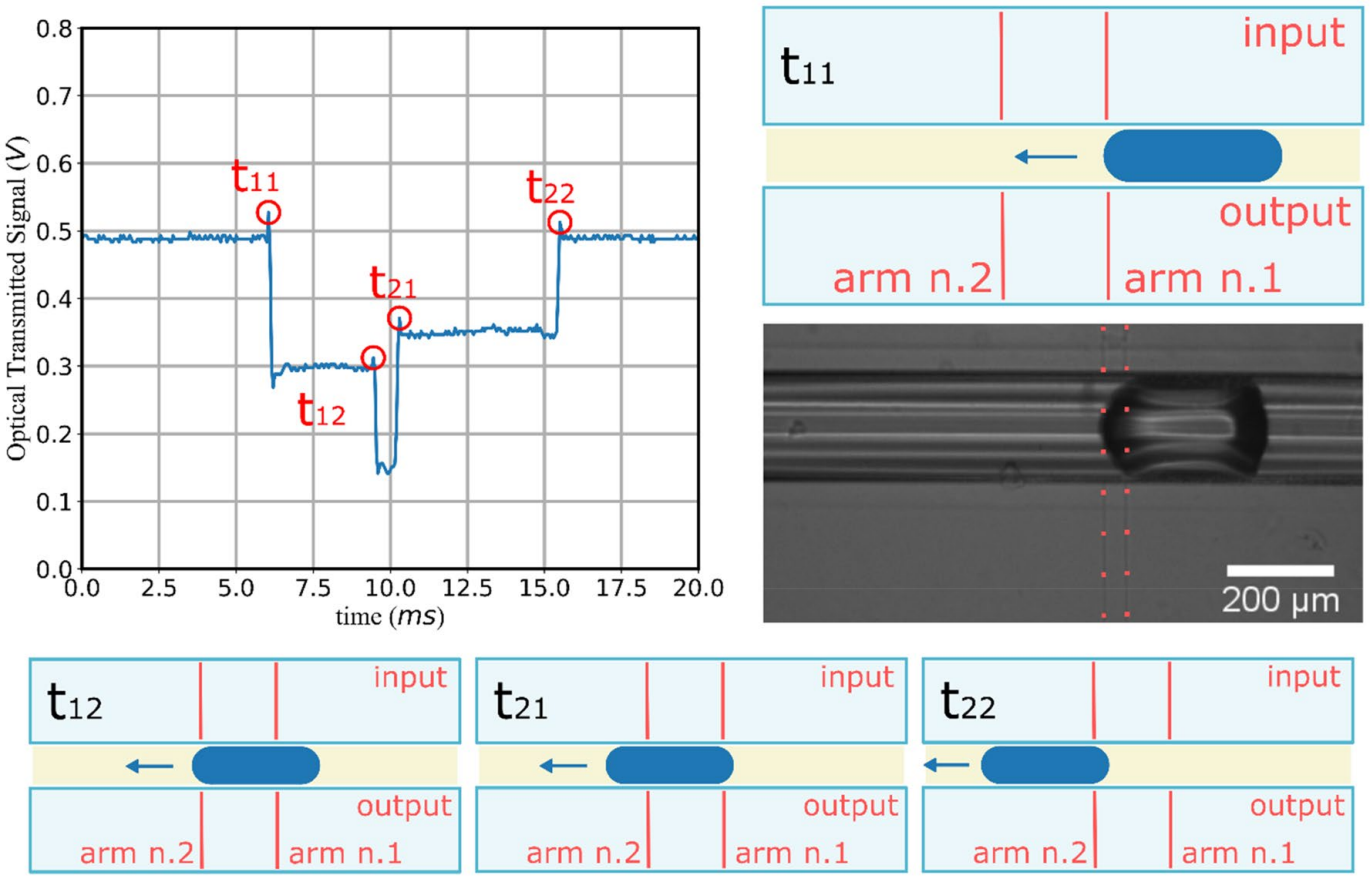
OT signal from the MZI. Example of the OT signal, where the trigger instants have been highlighted; the correspondence between the trigger instants and the droplet position with respect to the MZI waveguides is also illustrated. The picture is captured by the microscope system used as comparison, where the position of the MZI branches during the detection has been highlighted. The picture represents an example of a frame from the imaging system, highlighting the MZI branches during the detection.

The optical detection of the droplets has been made by measuring the intensity (OT signal) of the light exiting from the output waveguide, after it transmitted by the droplets. An example of the OT signal recorded when a single water droplet passes in front of the MZ waveguide is sketched in Fig. 3. Each time that a droplet interface crosses one branch of the MZI, the detected optical transmission changes depending on the refraction of light on the interface shape between the droplet and the surrounding continuous phase (oil). Therefore, the detection of the instants at which the OT signal changes (labelled as t_11,_ t_12,_ t_21,_ t_22_) allows to monitor the movement of droplet flowing inside the channel. In details, considering the Fig. 3:

- t_11_ refers to the start of the interaction between the droplet with the light transmitted across the first MZI arm and its transmission light has a drop, whereas the second arm still transmits,
- t_12_ represents the start of the interaction between the droplet with the light transmitted across the second MZI arm, and so both arms transmit low amount of light,
- t_21_ and t_22_ identify the two ends of the interaction between the droplet with both MZI arms, respectively.

These instants have been identified as the maximum before the drops (t_11_ and t_12_) and after the rises (t_21_ and t_22_) of the OT at each intensity changes (more details in Supplementary Information). Once these instants have been determined, the estimations of the length and velocity of each droplet are straightforward: the velocity of the droplet front and rear can be estimated as v_front_ = 2 W/(t_12_ − t_11_) and v_rear_ = 2 W/(t_21_ − t_22_), respectively. When droplets show comparable v_front_ and v_rear_, we considered their average v_average_ to determine the droplet lengths as L_1_ = (t_21_ − t_11_)v_average_ and L_2_ = (t_22_ − t_12_)v_average_.

Notably, the working principle of the MZI detection does not depend on the intensity of the optical transmission signal neither on the nature of the interaction, as long as the time instants mentioned above can be identified. Therefore, any liquids combinations can be detected as long as they provide a trigger signal which allows to distinguish the four instants between the different interactions of droplets with the two branches. Moreover, the tracking of a single droplet can be easily extended to the case of a sequence of droplets, whose movements can be monitored by considering the trigger times of each droplet and the corresponding OT value. Indeed, the detection and the labelling of a droplets sequence also relies on the different optical transmission values shown by the sequence of consecutive liquids interaction with the MZI. Specifically, three configurations can be settled up:

- if the water droplet crosses both MZI branches simultaneously, then the OT signal reaches a given value of transmission correlated with the droplet’s transmittivity (in the case of Fig. 3, water droplets have lower refractive than surrounding oil (continuous phase), therefore the transmission signal is minimum);
- if no droplets cross the MZI branches, then the OT signal is representative of the continuous phase optical transmission (Light for both branches propagate freely across the channel and light is not refracted by the droplets’ surface);
- if the droplet crosses only one branch at a time, then the OT signal assumes about half of the droplet transmittivity.

**Figure 4:**
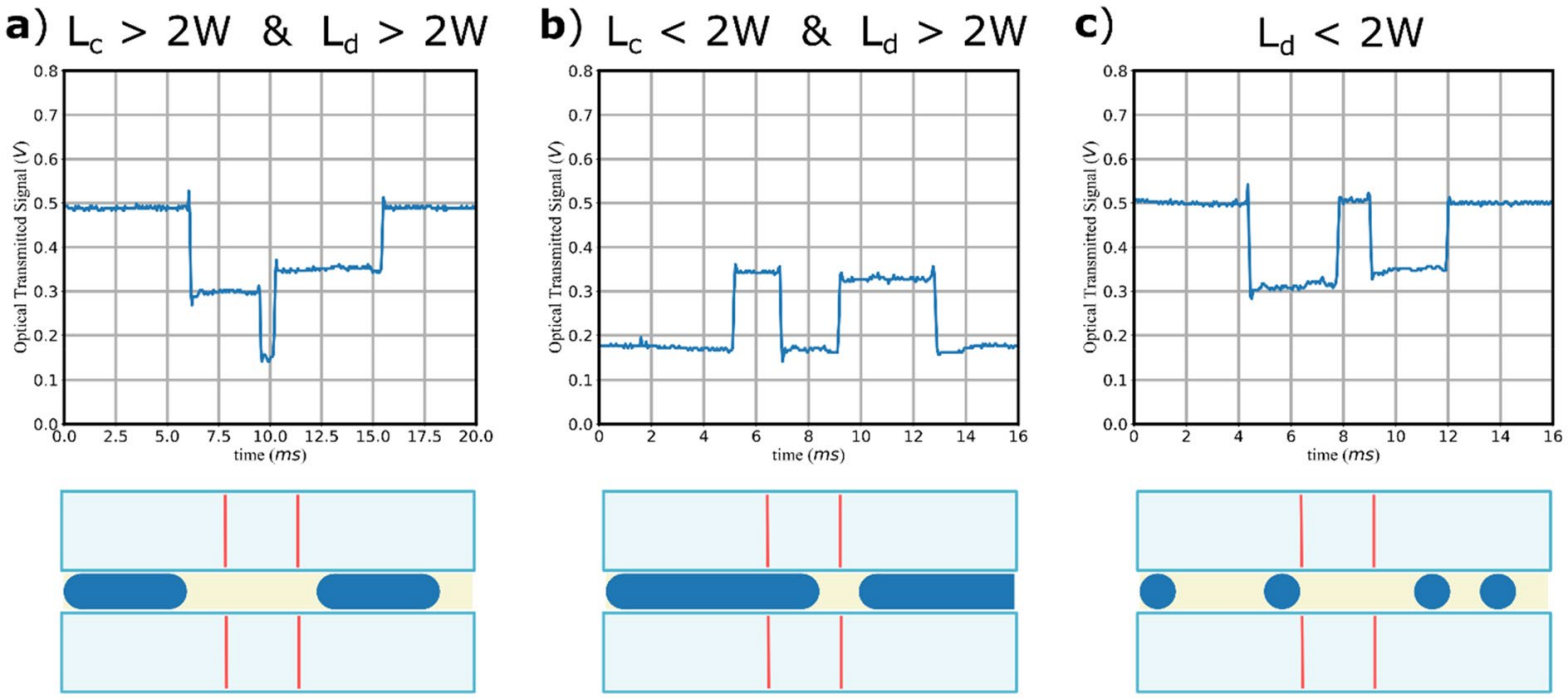
Detection regimes of droplets. Three possible regimes in terms of OT can occur when a droplet flows in front of the Mach Zehnder Interferometer, depending on the value of 2 W with respect to the length of the object L_c_ and the distance between two consecutive objects L_d_. (**a**) Both L_c_ and L_d_ are higher than 2 W, and therefore the droplet can interact with one or both the waveguided arms; (**b**) when L_c_ is smaller than 2 W but L_d_ is longer than 2 W, respectively always one or both the MZI branches are interacting with the droplet; (**c**) when both L_c_ and L_d_ are smaller than 2 W , the two branches cannot interact simultaneously with the same droplet. The measurements of the length and velocity can be made in all regimes just by triggering the timing of transition between the interaction situations.

Therefore, the OT signal from the MZI is a clear fingerprint of the droplets sequence and flow inside the channel, in a similar fashion of a recorded video form the imaging system. Moreover, depending on the distance between two consecutive droplet L_c_ and the length of the droplets L_d_, three possible regimes can be observed:

1. The first regime is characterized by L_c_ > 2 W and L_d_ > 2 W (Fig. 4a), and all the configuration a,b,c are observed;
2. The second regime is characterized by L_c_ < 2 W and L_d_ > 2 W (Fig. 4b), and only the configurations a,c are observed;
3. The third regime is characterized by L_d_ < 2 W (Fig. 4c), and only the situations b,c are observed. In terms of the OT signal, this regime shows similar feature for both situation of L_c_ < 2 W and L_c_ > 2 W. Indeed, the characterizing feature is that the configuration (a) is never observed for the same droplet.

In all the three regimes, the sequence of interactions between the droplets and the MZI can be clearly recognized, so the droplets sequence can be labelled by the OT signal. It is worth mentioning that also the detection of the velocity and length of each droplet is achieved in the same way, since t_11,_ t_12,_ t_21,_ t_22_ can be identified.

## Results and discussion

### Random droplets’ sequence univocal labelling

**Figure 5:**
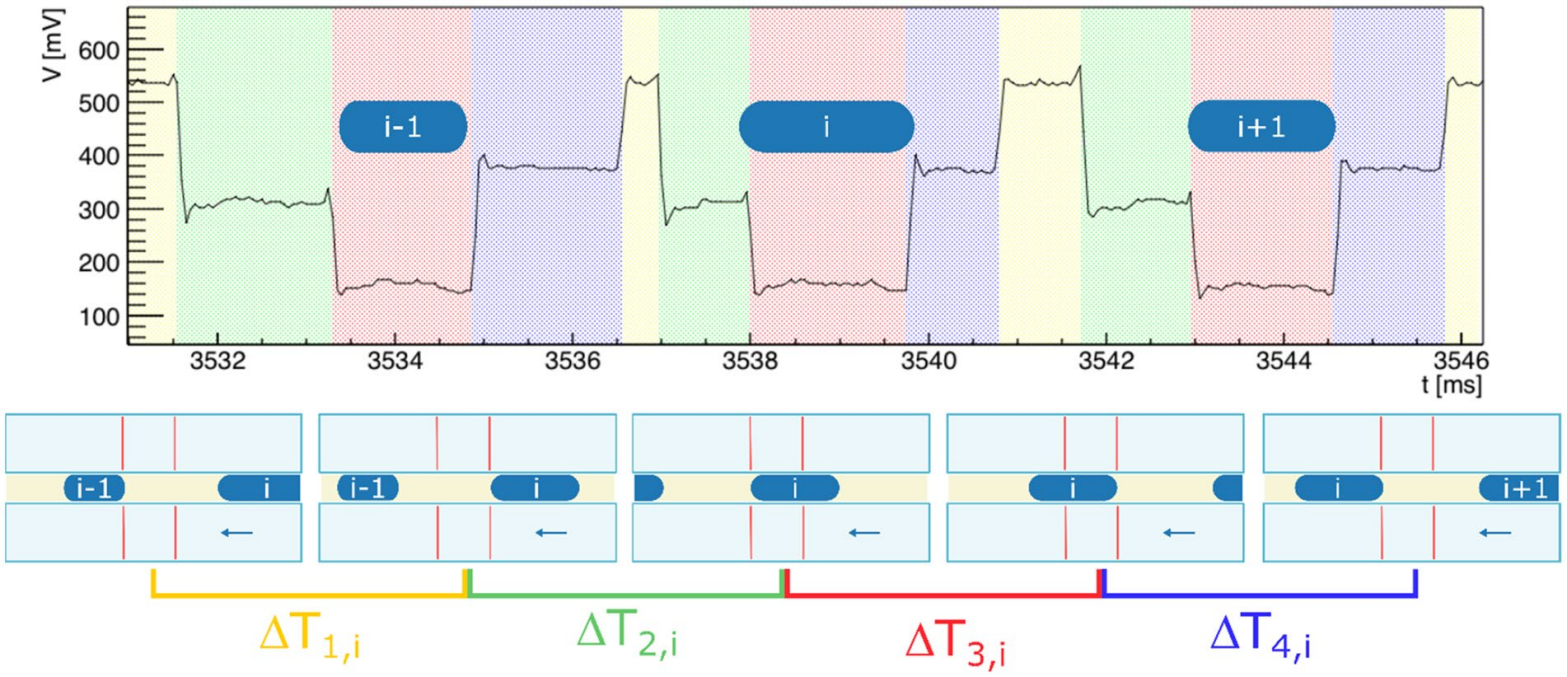
OT of three consecutive droplet, labeled as i − 1, i and i + 1. The four intervals of the i-droplet are called T_1i_, T_2i_, T_3i_ and T_4i_ respectively. The different configurations are highlighted by the colored regions: the red one refers to the situation (**a**), the light green and light blue to (**b**), and yellow to (**c**). Each of the four intervals are delimited by two specific instants when the droplet crosses the two branches, these intervals are sketched by the scheme below the graph.

The MZI configuration gives a univocally description of each droplet of a sequence, thanks to the fact that the trigger times of the OT signal can be associated to a well-defined configuration (a, b, c), the optofluidic device can be also employed to recognize and label sequence of droplets presenting arbitrary properties, both in shape or size. An example is reported in Fig. 5 the signal of three droplets generated with random size: depending on the size, different configurations are achieved providing for droplet shape-labelling. In the case the droplets differ in composition, the signal intensity changes accordingly providing also a composition-labelling. The labelling process can be easily achieved by monitoring the four time intervals ΔT_1_, ΔT_2_, ΔT_3_ and ΔT_4_, which are defined by the four trigger times (t_11_,t_12_,t_21_,t_22_) reported in Fig. 3. For instance, the i-droplet inside a sequence can be described as ΔT_1,i_ = [t_22_(of previous i − 1-droplet); t_11_], ΔT_2,i_ = [t_11_;t_12_], ΔT_3,ii_ = [t_12_;t_21_] and ΔT_4,1_ = [t_21_;t_22_] as shown in Fig. 5. The color boxes highlight the sequence of droplet passage between the two branches 1,2 and the three consecutive droplets with different lengths and velocities.

**Figure 6:**
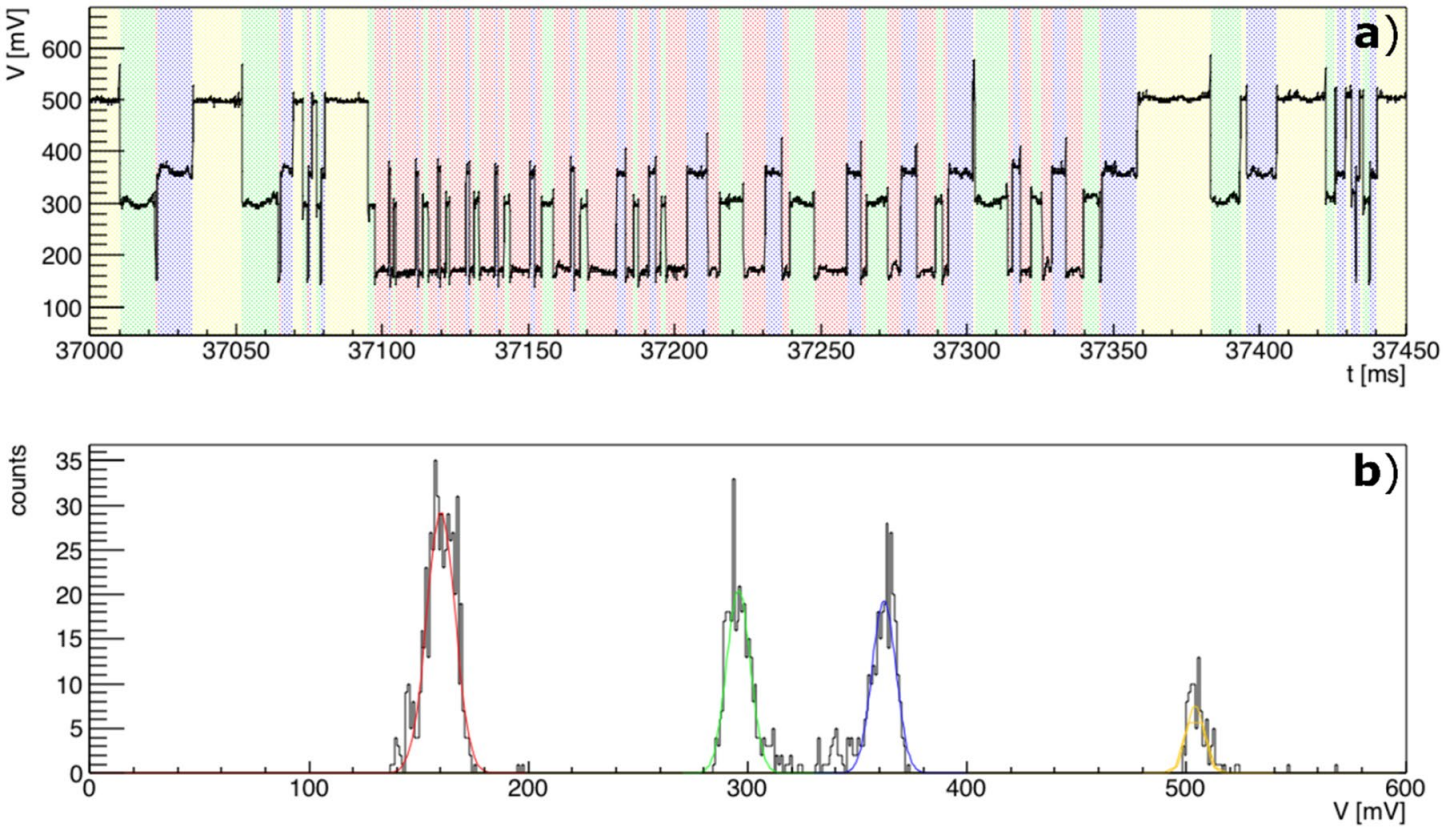
Labelling of a random and unknown sequence of droplets. (**a**): OT signal detected by the MZI during the flow of the 25 droplets, each of which is sequenced distinguishing each interval using colored boxes. The labelling evidenced four classes of OT signal data characterized by the histograms in (**b**), where each four time are fitted with gaussian function with corresponding color. The centroids of the gaussian are: 160 ± 0.3, 295.8 ± 0.4, 362 ± 0.4, 504 ± 0.6 mV and the standard deviation are: 6.5 ± 0.3, 5.4 ± 0.3, 5.4 ± 0.4, 4.0 ± 0.9 mV.

The labelling performances of the MZI has demonstrated to be particularly accurate and compatible with quantitative targeting procedures, where the identification of a dispersed phase (in this case a droplet, but easily applicable to bio-units like cells as described in the next section) is required together with its quantification. Figure 6a reports the signal of an arbitrary sequence of 25 droplets and the relative population distribution of the OT signal values using the sequencing feature described before (Fig. 6b). It should be clarified that the slight difference between the blue intervals and green ones in Fig. 6b is due to the slight asymmetrical behavior of the two arms of the MZI, due to variability in the fabrication process. Nevertheless, this difference does not influence the labelling process, which depends on the identification of difference between the value of these two values with the maximum and the minimum of the signal respectively.

The four intervals (ΔT_1,i_, ΔT_2,i_, ΔT_3,i_ and ΔT_4,i_) have been identified and labeled (using the same colors used in Fig. 5). The histograms of all the intensity of OT value have been fitted with a gaussian function providing a clear identification of four different peaks and the relative quantification. All the labelling data and results here reported have been checked and validated by the synchronous imaging acquisition (not reported) to assess the correct working principle of the MZI and verify that the labelling process was free of systematic errors. The validation process confirmed that the MZI platform can be used independently of the imaging acquisition with zero events of false positive/negative cases. The MZI labelling performance does not depend on the nature of the optical interaction between the analytes flowing inside a microfluidics channel. Notably, any combinations of liquids or also particle inside a liquid can be detected and labelled, as long as the optical interactions with the MZI provides an optical transmission difference between the relative ΔTs. A sequence of arbitrary droplets flowing inside the channel can be successfully monitored by the OT signal detecting the subsequent ΔTs. The accuracy of the labelling process depends on the capability to distinguish the four plateaus, and in particular the maximum and the minimum from the medium ones. This depends on the nature of the optical interaction between the sample on the MZI and the waveguided beam, whereas the width of their distribution depends on the fluctuations of the system. We observed a maximum standard deviation of 6.5 mV of the distributions. Therefore, the accuracy of the labelling depends on the confidence level to distinguish two plateaus (minimum and maximum vs medium ones), which is related to the latter value. In the case of air-in-oil droplets with a difference of refractive index of Δn = 0.434, we observed a minimum difference between the plateau of 135.8 ± 0.5, which is more than 20 times the maximum standard deviation of the distribution. As long as the difference is higher than 5 times, the accuracy of the labelling process of less than 10^6^ droplets can be considered 100%.

### Detection of single droplets and emulsions

The MZI platform has been widely tested to assess its performance as velocity and length sensor. It has been therefore compared to the standard imaging system technique, both for the case of a single droplet and for the emulsions (i.e. sequence of droplets generated with same flowrates). Considering the simultaneous detection of the same droplet by the MZI and the microscope, the two techniques show compatible results, presenting a maximum discrepancy of almost 1%. The uncertainty of the two methods are estimated by the standard deviation of the detection of the same droplet over more than 10 frames for the imaging system, and by the acquisition frequency and the uncertainty on 2 W of the MZI system. For droplets length in the range [229:626] μm, the detection of the imaging method presents an uncertainty between 0.6 and 1.7%, whereas the one of the MZI varies between 0.11 and 1.6%, thus showing comparable performances of the two approaches. Similarly, the velocity detection (droplet velocity in the range [7.9:59] μm/ms), the imaging system shows an uncertainty of 0.05–0.015%, and the MZI between 0.001 and 0.013%.

To extend the applicability of the of device also to the case of a sequence of droplets rapidly flowing inside the microfluidic circuit, 43 emulsions of more than 100 droplets each have been produced varying the flowrates of the two phases as reported in Table 1.

**Table 1:** Datasets of the emulsion. Three main datasets have been tested: two with fixed flowrates of the dispersed phase, and one with fixed ratio between the two flowrates used for the droplets production. The flowrate of the continuous phase Q_c_ is considered as the sum of the flowrate injected in the two channels of the cross-junction.

Number of emulsions	Flowrate of the continuous phase Q_c_ (µL/min)	Flowrate of the dispersed phase Q_d_ (µL/min)	Length range (μm)	Velocity range (μm/min)	Label of the set
24	[10,15,20,…,125]	10	229–626	7.9–59	Q_d_ = 10
10	[10,…,90]	20	287–589	11.3–40.7	Q_d_ = 20
9	[10,15,20,…,55]	[10,15,20,…,55]	589–621	11.8–44.3	ϕ = Q_c_/Q_d_ = 1

**Figure 7:**
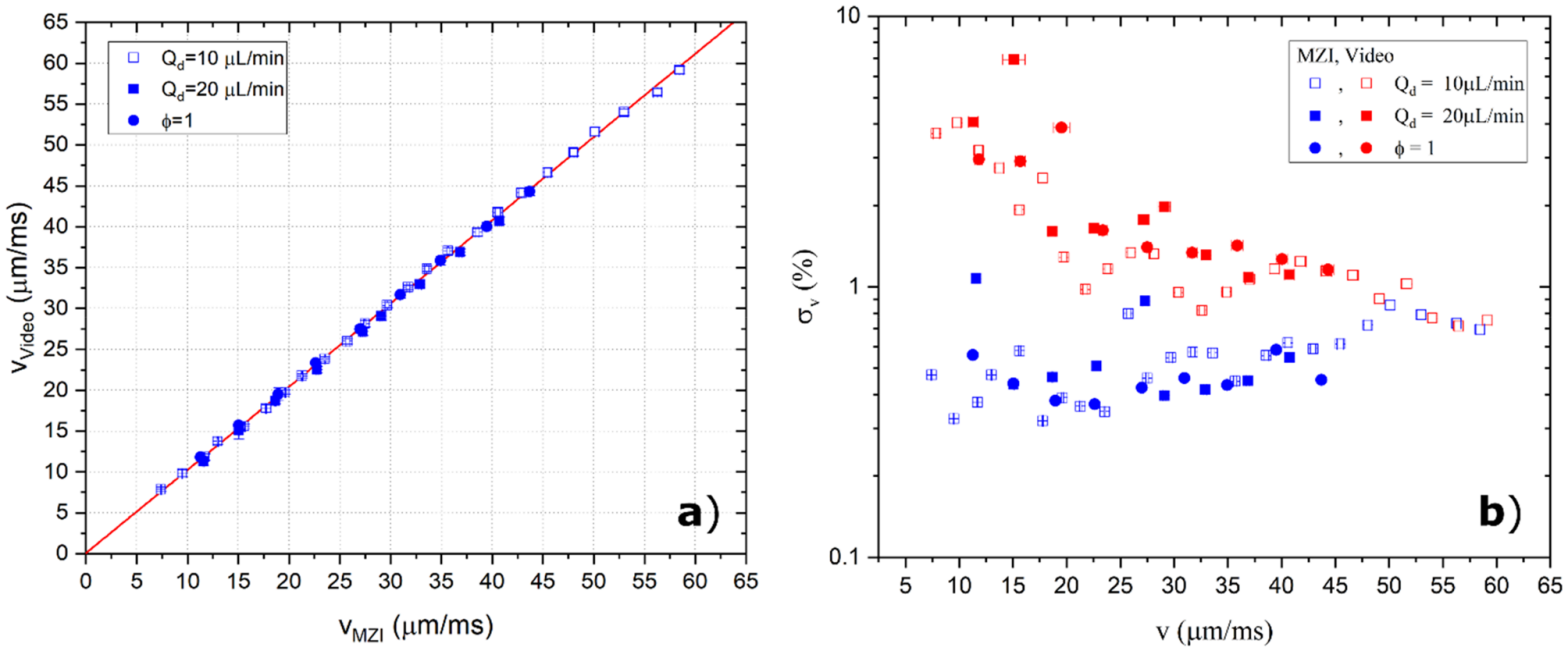
Comparison between the two data acquisition systems (the MZI and the Video) for the emulsion detection. Figure 5a reports the mean of the velocity distribution of each emulsion comparing the one obtained with the Video (in y) and the one obtained with the MZI (in x). The data are labelled distinguishing the three different data sets: Q_d_ constants at 10 and 20 µL/min, Φ = Q_d_/Q_c_ = 1. The linear interpolation (red line) shows an intercept of 0.1 ± 0.2 µm/ms and a slope of 1.018 ± 0.005. Figure 5b reports the variation of the standard deviations of the velocity distribution of each emulsion over the range of the velocity tested. The data are reported in % with respect to average of the relative distribution and in the log scale. Blue points refer to the MZI distributions and red points to the Video ones, whereas the different markers label the different datasets.

For every emulsion, the detection of the velocity and length of each droplet have been performed simultaneously with standard microscope and fast camera system (“Video” at least 10 frames for each droplet) and the optical waveguide in MZI configuration (“MZI”). For both the two parameters, the average and the standard deviation of their distribution is considered. As a way of example, a complete overview for the velocity measurements is reported in Fig. 7, where the means of the distribution (Fig. 7a) and the standard deviations (Fig. 7b) are compared in order to test the accuracy of the MZI. In Fig. 5a, the slope of the linear regression (red line with R^2^ > 0.999) is 1.018 ± 0.005 thus suggesting a high degree of compatibility of the detection process performed with the two methods over all the range tested. Similarly, the analysis for the droplet length provides a slope of 0.996 ± 0.008 with a R^2^ > 0.995. Moreover, Fig. 5b reports the value of the standard deviation of the velocity distribution (normalized by the mean of the distribution), which depends on both the precision of the detection systems used for the measurements and on the dispersion of the emulsion produced by the droplet generator. Since the measurement made by the two systems are simultaneous, graph Fig. 5b gives a direct comparison of the uncertainty between the two detection methods. Notably, the MZI reports dispersions of the velocity of the emulsion always lower than 1% and lower than the imaging system. The same results are obtained for the droplet lengths detection.

It is worth mentioning that the detection relies only on small power light source and simple light detectors as photodiodes, since the losses of the waveguide are 3 ± 1 dB cm^−1^^35^ and the transmission across the channel is about 70%^36^. Furthermore, integrated optical waveguide provides a complete portability of the system, that can be pigtailed with any optical fiber coupled lasers^53^, thus ensuring an optimal alignment of the light coupling with the channels. Several waveguides have been previously demonstrated in various material, such as glass^32^, PDMS^31^ or SU8^30^, where the MZI could be integrated using the same detection protocol. Nevertheless, the integration in lithium niobate can be exploited for combining MZI in the same platform with its outstanding capability in droplets manipulation, such as photovoltaic tweezers^46,47^ or SAW system^44,45^, thus making straightforward the integration of the MZI with droplet generators and droplets manipulation for lab-on-a-chip applications using the same monolithic platform of lithium niobate.

Besides the clear advantageous integrability of the detection system, the MZI system showed also a competitive sensitivity in both velocity and length detection compared to standard video analysis system. An uncertainty of less than 1% on the detection of both single droplet velocity and length, as well as emulsion, makes the MZI more precise than standard imaging detection. Furthermore, a rise of sensitivity can be further achieved by an increase of the acquisition frequency of the DAQ, whereas similar sensitivity gain in standard system can be obtained by improving the frame rates of the fast camera. Nevertheless, the cost and the bulkiness of improved fast camera hinders drastically the performances, whereas DAQ improvement can be easily achieved. Additionally, the higher frames records rise drastically the post processing operation times and the storage of the raw signal (usually Gbs), whereas the voltage OT signal of the MZI can be easily processed in real-time and it can be used easily for feedback approaches.

### Comparison with other methods

The standard integrated system are based on the measurements of the time passage of droplets^14,20,23,27^, which depends on both velocity and length. This feature hinders not only the detection of the two properties but also any labelling and sequencing capability. In fact, the presence of two detecting points give the additional potentiality of detecting velocity and length in a uncorrelated way, for instance by the two detector system proposed by Hsieh^24^ and by Hassan^25^. The MZI combined such potentiality provided by two points reference with the integrability of a single detection system in the same method, providing also the sequencing of droplets. In addition, the MZI geometry is commonly integrated in microfluidic devices for sensing purpose exploiting its interferometric sensitivity, where only one arm is used as a reference, and its integration process is well-known. Therefore, the exploitation of this structure for velocity and size droplets detection enables the unique feature of single optical system still using two points references, thus allowing multiplexing of two systems into a single one.

Contrary to the detectors based on two optical fibers, the feasibility of a waveguided MZI structure allows also for a high potential scaling power of the system. Indeed, the device can be reduced in size without limitations in terms of width of the channel, thus achieving the detection of smaller objects (down to few µm), as required for instance in the detection of biological-units. This feature cannot be obtained with standard fiber-based devices, due to the groove for the fiber embedding. The only limitation in terms of size range and scaling is the independent detection of the two points reference. In the MZI structure, this is related to the 2 W distance and the diffraction of the light across the channel (further details on Supplementary Information). In particular, for the device here presented (channel width of 200 µm) 2 W should be higher than 26 µm, which limits the detection of droplet in the first regimes of 26 µm. In principle, the limit of detection depends simply on the size of the waveguide, which is much more confined than an optical fiber. In our case, the main guided optical mode has a full width at half maximum of 2.8 µm. The velocity detection range can be further extended improving the electronics for data acquisition. For the data reported here the maximum acquisition frequency was 200 kHz, which corresponds to a maximum velocity 8 ms^-1^.

Furthermore, the MZI configuration does not require any calibration, that instead is needed in most of the electrical-based methods. The MZI platform is extremely stable and durable, highly resistant to chemical attack thanks to the LiNbO_3_ properties. This represents an added value respect the majority of other platforms that are mostly based on the embedded fibers to couple light and the presence of grooves. Local change in the morphology of the channel has been there reported, for instance as showed in the device presented by Hsieh^24^. Instead in this work, the use of waveguide is crucial for a undisturbed flows of the droplets during detection. Besides these considerations, the ranges tested and the sensitivity of the detection makes the presented device outstanding in the performances for both the velocity and length, as show in Table 2.

**Table 2:** Comparison between different integrated for the measurements of lengths and velocity of flowing droplets.

Technique	Features	Ranges and precision
Thermal^14^	Only counting	−
Impedimetric^15^	Both velocity and length detection	Length > 100 μm Uncertainty: 12.70% and 7.61% for velocity and length respectively
Capacitative^20^	Only velocity and require calibration	15 Hz droplet frequency Uncertainty: 5%
Microwave^22^	Only emulsion detection, no velocity	Length: 480–310 μm uncertainty: 10% droplets frequency: 2.6–8 droplets/s
Optical Fiber^23^	Only emulsion detection. Requires fiber integration	No mention about uncertainty
Optical fiber^24^	Both velocity and length. Require fiber integration and two detectors	Length: 1.03–5.05 mm, velocity: 1.94–71.5 mm/s Uncertainty: < 3.4% for both length and velocity
Optical Fiber^29^	Both velocity and length. Require fiber integration	Length: 15–60 µm flow velocity: 3.5 mm/s-5.0 mm/s Uncertainty: 10% for emulsion length, 2% for the frequency
Optical Flow cell^25^	Both velocity and length. No integration inside microfluidic channel	Length > 800 µm velocity: 0.2–5.45 mm/s Uncertainty: 6% on velocity estimation < 5% on length
MZI waveguides	Both velocity and length detection. Single droplet, emulsion and random sequence labelling	Length: 229–626 µm velocity: 7.9–59 mm/s Uncertainty: < 1% for both velocity and length

## Conclusions

This paper presents an integrated optofluidic chip for labeling, sequencing and detecting lengths and velocities of micro-droplets. It consists of an Integrated Mach–Zehnder (MZI) waveguides configuration orthogonally crossed by a microfluidic channel. The detection is based on the interaction between the droplet flowing inside the microfluidic channel and the light guided in the MZI, since the passage of a droplet in front of the two MZI branches leads to a modulation of the optical signal transmitted across the fluidic channel. The sequencing and detection of the droplets as well as the measure of their length and velocity are obtained by triggering the instants at which the intensity of the output optical signal changes due to the interaction between the droplets and the waveguided light. Since the method is based on the temporal analysis of the signal, in principle the intensity signal can be modulated by any optical interactions, thus opening new perspective for a wide range of analytes, such as biological samples like cell or particles. The system has been recently demonstrated to be suitable for measuring also optical properties inside the microfluidic channel, such as optical absorbance^35^. In addition, the detection there presented does not requires any specific optical wavelength of the light source neither optical interaction, thus making it suitable for Fluorescence application or other sensing applications related optical properties detection.

In this work, the MZI based configuration is coupled with a Cross-junction droplets generator in a lithium niobate substrate, which is exploited for testing emulsion. The comparison of MZI and standard imaging system is carried out by analyzing water in oil droplets with velocities between 7.9 and 59 μm/ms and lengths between 229 and 626 μm. MZI showed a reliable detection of both velocity and length of single droplet and of droplets sequences, in comparison with simultaneous detection made by standard imaging method. For both values, the device demonstrated uncertainty lower than 1%, and lower than competitors. Furthermore, the simplicity given by the optical signal leads to real-time processing, to immediate response time and to an improvement of storage consumption. It is also worth mentioning the integration level of the waveguided structure, which can be easily pigtailed with fibers, ensuring high reproducibility for different device and high portability without hindering the performances. Finally, such sequencing and detecting system integrated in lithium niobate substrate could pave the way for multifunctional lab-on-a-chip, in combination with the outstanding and unique properties of this material for micro- and nano-manipulation.

## Supplementary Information

Supplementary Information.

## References

[1] Whitesides GM (2006). The origins and the future of microfluidics. Nature.

[2] Teh SY, Lin R, Hung LH, Lee AP (2008). Droplet microfluidics. Lab Chip.

[3] DeMello AJ (2006). Control and detection of chemical reactions in microfluidic systems. Nature.

[4] Guo MT, Rotem A, Heyman JA, Weitz DA (2012). Droplet microfluidics for high-throughput biological assays. Lab Chip.

[5] Joensson HN, Andersson Svahn H (2012). Droplet microfluidics-A tool for single-cell analysis. Angew. Chem. Int. Edn..

[6] Kaminski TS, Scheler O, Garstecki P (2016). Droplet microfluidics for microbiology: Techniques, applications and challenges. Lab Chip.

[7] Zhang, Y. & Jiang, H.-R. A review on continuous-flow microfluidic PCR in droplets: Advances, challenges and future (2016). 10.1016/j.aca.2016.02.006. 10.1016/j.aca.2016.02.006 26965323

[8] Song, H., Chen, D. L., Ismagilov, R. F. & Ismagilov, R. F. Droplet-Based Microfluidics Reactions in Droplets in Microfluidic Channels Angewandte Chemie Keywords: analytical systems · interfaces · microfluidics · microreactors · plugs Reviews 7336. 10.1002/anie.200601554.

[9] Zhu P, Wang L (2017). Passive and active droplet generation with microfluidics: a review. Lab Chip.

[10] Jakiela S, Makulska S, Korczyk PM, Garstecki P (2011). Speed of flow of individual droplets in microfluidic channels as a function of the capillary number, volume of droplets and contrast of viscosities. Lab Chip.

[11] De Saint Vincent MR, Cassagnére S, Plantard J, Delville JP (2012). Real-time droplet caliper for digital microfluidics. Microfluid. Nanofluidics.

[12] Basu AS (2013). Droplet morphometry and velocimetry (DMV): A video processing software for time-resolved, label-free tracking of droplet parameters. Lab Chip.

[13] Chong ZZ (2016). Automated droplet measurement (ADM): an enhanced video processing software for rapid droplet measurements. Microfluid. Nanofluidics.

[14] Yi N, Park BK, Kim D, Park J (2011). Micro-droplet detection and characterization using thermal responses. Lab Chip.

[15] Saateh A (2019). Real-time impedimetric droplet measurement (iDM). Lab Chip.

[16] Moiseeva EV, Fletcher AA, Harnett CK (2011). Thin-film electrode based droplet detection for microfluidic systems. Sensors Actuators B Chem..

[17] Fu H, Zeng W, Li S, Yuan S (2017). Electrical-detection droplet microfluidic closed-loop control system for precise droplet production. Sensors Actuators A Phys..

[18] Dong T, Barbosa C (2015). Capacitance variation induced by microfluidic two-phase flow across insulated interdigital electrodes in lab-on-chip devices. Sensors (Switzerland).

[19] Niu X, Zhang M, Peng S, Wen W, Sheng P (2007). Real-time detection, control, and sorting of microfluidic droplets. Biomicrofluidics.

[20] Elbuken C, Glawdel T, Chan D, Ren CL (2011). Detection of microdroplet size and speed using capacitive sensors. Sensors Actuators A Phys..

[21] Lombardo T, Lancellotti L, Souprayen C, Sella C, Thouin L (2019). Electrochemical detection of droplets in microfluidic devices: Simultaneous determination of velocity, size and content. Electroanalysis.

[22] de Novais Schianti J (2019). Novel platform for droplet detection and size measurement using microstrip transmission lines. Sensors (Basel)..

[23] Nguyen NT, Lassemono S, Chollet FA (2006). Optical detection for droplet size control in microfluidic droplet-based analysis systems. Sensors Actuators B Chem..

[24] Hsieh YW, Wang AB, Lu XY, Wang LA (2016). High-throughput on-line multi-detection for refractive index, velocity, size, and concentration measurements of micro-two-phase flow using optical microfibers. Sensors Actuators B Chem..

[25] Hassan SU, Nightingale AM, Niu X (2017). Optical flow cell for measuring size, velocity and composition of flowing droplets. Micromachines.

[26] Hassan S. U, Nightingale AM, Niu X (2018). Micromachined optical flow cell for sensitive measurement of droplets in tubing. Biomed. Microdevices.

[27] Bettella G (2019). LiNbO3 integrated system for opto-microfluidic sensing. Sensors Actuators B Chem..

[28] Kunstmann-Olsen C, Hanczyc MM, Hoyland J, Rasmussen S, Rubahn HG (2016). Uniform droplet splitting and detection using Lab-on-Chip flow cytometry on a microfluidic PDMS device. Sensors Actuators B Chem..

[29] Shivhare PK, Prabhakar A, Sen AK (2017). Optofluidics based lab-on-chip device for in situ measurement of mean droplet size and droplet size distribution of an emulsion. J. Micromech. Microeng..

[30] Mogensen KB, El-Ali J, Wolff A, Kutter JP (2003). Integration of polymer waveguides for optical detection in microfabricated chemical analysis systems. Appl. Opt..

[31] Kee JS, Poenar DP, Neuzil P, Yobas L (2008). Monolithic integration of poly(dimethylsiloxane) waveguides and microfluidics for on-chip absorbance measurements. Sensors Actuators, B Chem..

[32] Nava G (2015). All-silica microfluidic optical stretcher with acoustophoretic prefocusing. Microfluid. Nanofluidics.

[33] Vitali V (2020). Integrated optofluidic chip for oscillatory microrheology. Sci. Rep..

[34] Bonfadini S (2019). Optofluidic platform using liquid crystals in lithium niobate microchannel. Sci. Rep..

[35] Zamboni R (2020). Opto-microfluidic system for absorbance measurements in lithium niobate device applied to ph measurements. Sensors (Switzerland).

[36] Fares L. Al, Devaux F, Guichardaz B, Chauvet M (2013). Self-trapped beams crossing tilted channels to induce guided polarization separators. Appl. Phys. Lett..

[37] Balslev S (2006). Lab-on-a-chip with integrated optical transducers. Lab Chip.

[38] Wooten EL (2000). Review of lithium niobate modulators for fiber-optic communications systems. IEEE J. Sel. Top. Quantum Electron..

[39] Bazzan M, Sada C (2015). Optical waveguides in lithium niobate: Recent developments and applications. Appl. Phys. Rev..

[40] Vittadello L (2016). Photorefractive direct laser writing. J. Phys. D. Appl. Phys..

[41] Ferraro P, Coppola S, Grilli S, Paturzo M, Vespini V (2010). Dispensing nano-pico droplets and liquid patterning by pyroelectrodynamic shooting. Nat. Nanotechnol..

[42] Coppola S, Vespini V, Grilli S, Ferraro P (2011). Self-assembling of multi-jets by pyro-electrohydrodynamic effect for high throughput liquid nanodrops transfer. Lab Chip.

[43] Vespini V, Coppola S, Grilli S, Paturzo M, Ferraro P (2011). Pyroelectric adaptive nanodispenser (PYRANA) microrobot for liquid delivery on a target. Lab Chip.

[44] Lin SCS, Mao X, Huang TJ (2012). Surface acoustic wave (SAW) acoustophoresis: Now and beyond. Lab Chip.

[45] Zhang SP (2018). Digital acoustofluidics enables contactless and programmable liquid handling. Nat. Commun..

[46] Esseling M, Zaltron A, Horn W, Denz C (2015). Optofluidic droplet router. Laser Photonics Rev..

[47] Jubera M, Elvira I, García-Cabañes A, Bella JL, Carrascosa M (2016). Trapping and patterning of biological objects using photovoltaic tweezers. Appl. Phys. Lett..

[48] Zaltron, A. *et al.* Integrated optics on Lithium Niobate for sensing applications. In *Optical Sensors 2015* (2015). 10.1117/12.2178457.

[49] Bettella G (2017). Lithium niobate micromachining for the fabrication of microfluidic droplet generators. Micromachines.

[50] Lucchetti L, Kushnir K, Zaltron A, Simoni F (2016). Light controlled phase shifter for optofluidics. Opt. Lett..

[51] Bettella, G. *et al.* Integrated opto-microfluidics platforms in lithium niobate crystals for sensing applications. In *Integrated Optics: Devices, Materials, and Technologies XIX* (2015). 10.1117/12.2077843.

[52] Langelier SM, Yeo LY, Friend J (2012). UV epoxy bonding for enhanced SAW transmission and microscale acoustofluidic integration. Lab Chip.

[53] Keil R, Auracher F (1979). Coupling of single-mode Ti-diffused LiNbO3 waveguides to single-mode fibers. Opt. Commun..

