# Supplementary material for: Real-time precise microfluidic droplets label-sequencing combined in a velocity detection sensor
Source: Sci Rep. 2021 Sep 9;11:17987. doi: 10.1038/s41598-021-97392-3 (PMC8429775; doi:10.1038/s41598-021-97392-3)
Supplement: Supplementary file 1 — Supplementary Information. [file 41598_2021_97392_MOESM1_ESM.docx]

**Real-time precise microfluidic droplets label-sequencing combined in a velocity detection sensor.**

R. Zamboni^1,2^, A. Zaltron^1^, M. Chauvet^3^ and C. Sada^1,^*

1. University of Padua, Physics and Astronomy Department, Via Marzolo 8, 35131 Padova, Italy;

2. [Institute of Applied Physics, University of Münster](https://aip.scitation.org/action/doSearch?field1=Affiliation&text1=Institute%20of%20Applied%20Physics,%20University%20of%20M%C3%BCnster&field2=AllField&text2=&Ppub=&Ppub=&AfterYear=&BeforeYear=&access=), Corrensstrasse 2/4, 48149, Münster, Germany;

3. University of Bourgogne Franche-Comté, FEMTO-ST Institute, UMR 6174, 15B Avenue des Montboucons, 25000 Besançon, France;

Corresponding author: [cinzia.sada@unipd.it](mailto:cinzia.sada@unipd.it) (C.S.); Tel.: +39-049-8277037,

riccardo.zamboni@phd.unipd.it (R.Z.);

annamaria.zaltron@unipd.it (A.Z.);

mathieu.chauvet@univ-fcomte.fr (M.C.).

**Supplementary Information:**

**Mach-Zehnder design and Ti diffusion:**

The design of the integrated Mach-Zehnder is realized considering that both branches must interact independently with the droplets inside the channel. For this reason, it is needed to set the right values for 2W, the distance between the two branches of MZI, (see Fig. 1) in order to prevent a cross-coupling of the light coming from the arm n.1 of the input into the output waveguide arm n.2, due to a wider light cone’s aperture respect to the arms distances. The cone aperture is described in terms of Numerical aperture (NA) and depends on both the dispersion angle θ and the refractive index n_L_ of the medium fluxing inside the channel, as NA = n_L_ sin(θ). In particular, by defining the width of the micro-fluidic channel as w and being d the light beam waist at the end of the channel width, it results that d = w tan(θ). For *z*-propagating Ti-diffused waveguides in lithium niobate, the numerical aperture can be estimated as NA_eff_ = 0.13 and the maximum d value obtained in case of air medium is equal to d_air_ = 13.2 μm for a channel of w = 100 μm. In this case, 2W is designed to be higher than d_air_, in order to ensure the complete independency between the two MZI branches. In the present configuration, the optimal design was settled with 2W = 40 μm.


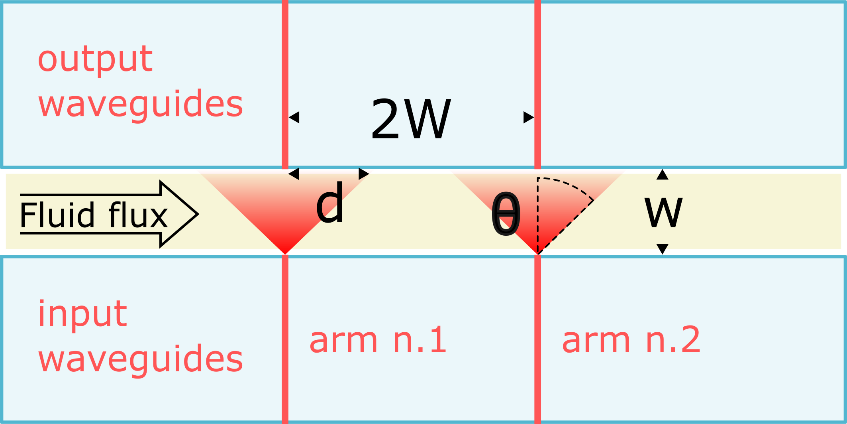


**Figure 1 Scheme of the two MZI branches output inside the channel.** Two cones of light are dispersed inside the channel described by the angle θ and NA.

2W has been measured by near-field technique using a long working distance 50x/0.55 objective, collecting the light of the two spots of the input waveguides. Fig. 2 reports the CCD picture of the two spot. The average over several MZIs reports 2W = (42±1) μm.


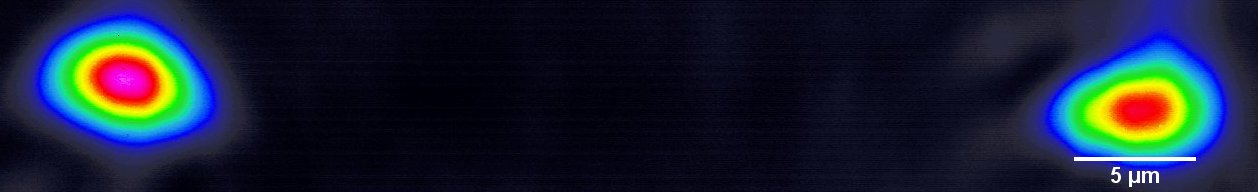


**Figure 2: Near-field image of the emerging light from a MZI waveguided structure in the proximity of microfluidic channel**. The color is not the real output but are used to shows the intensity distributions.

The design of the other parts composing MZI structure have been studied in order to achieve maximum transmission: the two Y-junction and the tapered region, which connects and split the single waveguide to the two parallel branches are designed to maximize the transmission properties.

The optimal design^1^ for the tapered region of a single mode waveguide (considering a maximum size of the Y of 0.3 mm) is a tapered region of angle 0.5°. The angle of the Y is 2.8° and two S-section connects the Y-junction to the straight section, realized with two arc of circle with the same radius of curvature of 12.5 mm. The straight arms section is designed to be 8 mm to enable an good alignment of the microchannel during the engraving of the channel. The length of the complete MZI structure (from the first Y to the end of the second) between the single straight waveguides is 9.6 mm. As described by Baets^1^, this design ensure optical losses lower than −0.5 dB.

| 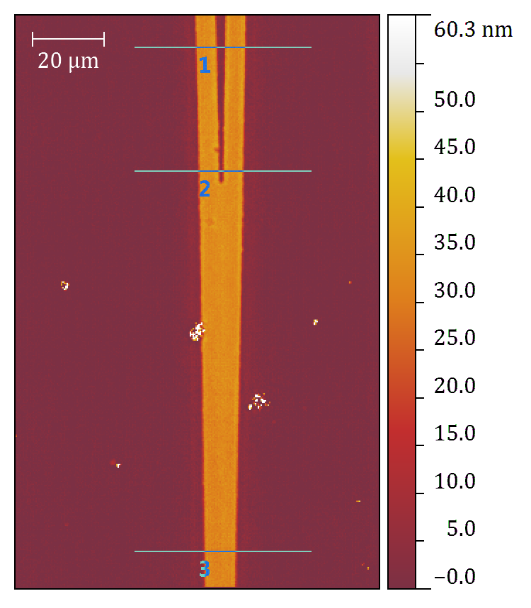 | 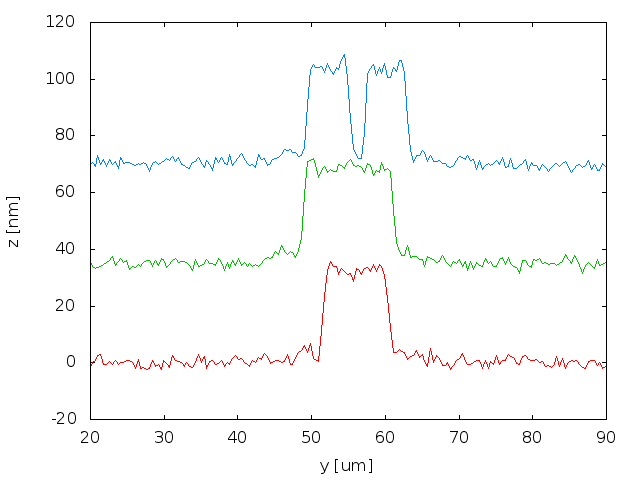 |
| --- | --- |
| **a**) | **b**) |

**Figure 3: AFM analysis of the Ti layer at the Y-junction.** a) report the 2D map of the AFM analysis, b) three profile (shifted in the z axis for visualization purpose) across the Y-junction.

The Titanium stripes with the pattern before mentioned were realized by photolithographic technique using positive photoresist S1805 from the S1800 Series (Dow Chemical). Before the photoresist depositions, the substrates were coated with hexamethyldisilizane (HDMS, Dow Chemical) based primer at 2000 rpm for 30 s. Then, the S1805 was spinned at 6000 rpm for 60 s. The layer thickness was measured with a point contact profilometer P-10 (KLA Tencor), and the results was 0.5 ± 0.1 μm , compatible with the value given by the supplier. Before the UV exposure, the sample with the photoresist was soft baked at 60−80° for 10 min. Then, the samples were exposed at 9 mW cm^−2^ of UV light for 18 s and right after, another bake step was done at 80° for 2 min. The developments were performed by means of soak for 60 min into the developer microposit MF-319 (Dow Chemical). The following process was the Ti deposition, made by a magnetron-sputtering machine (Thin film technology) in Argon atmosphere at 5 × 10 ^−3^ mbar, with a DC power of 40 W for 22 min. The resulting Ti-layer had a thickness of 30 nm measured by means of AFM (Veeco CP-II). The samples with photoresist and Ti layers were finally dipped into a solution of SVC(TM)-14 (Dow Chemical) at a temperature of 60 °, until the photoresist was completely removed from the sample. Before diffusion, the structure was checked by AFM. Fig. 3 reports an AFM analysis of the Ti layer at the Y-junction. The latter shows a resolution of 0.48 μm respect to the original design, and it is compatible with the one suggested by the photoresist producer.

**Microchannel design and droplet production:**

The droplet emulsion have been produced by a cross-junction configuration as depicted in Fig. 4. All three channel are 200 μm wide and 100 μm deep. The flow rates of both the two channel of the continuous phase has been fixed to Q_c_/2.


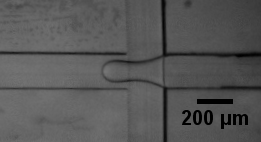


**Figure 4: Droplet formation in Cross-junction.** The picture shows the water droplet formation in lithium niobate cross-junction.

The device performances have been tested on sequences of droplets rapidly flowing inside the microfluidic circuit, 43 emulsions of more than 100 droplets each have been produced varying the flowrates of the two phases as reported in Tab. 1.

**Table 1: Datasets of the emulsion.** Three main datasets have been tested: two with fixed flowrates of the dispersed phase, and one with fixed ratio between the two flowrates used for the droplets production. The flowrate of the continuous phase Q_c_ is considered as the sum of the flowrate injected in the two channels of the cross-junction.

| Number of emulsions | Flowrate of the continuous phase Q_c_ (µL/min) | Flowrate of the dispersed phase Q_d_ (µL/min) | Length range  (μm) | Velocity range  (μm/min) | Label of the set |
| --- | --- | --- | --- | --- | --- |
| 24 | [10,15,20,…,125] | 10 | 229-626 | 7.9-59 | Q_d_ = 10 |
| 10 | [10,…,90] | 20 | 287-589 | 11.3-40.7 | Q_d_ = 20 |
| 9 | [10,15,20,…,55] | [10,15,20,…,55] | 589-621 | 11.8-44.3 | φ (= Q_c_/Q_d_) =1 |

The wettability on the channel surfaces has been homogenize using a Self Assembled Monolayer of Octadecyltrichlorosilane (OTS, Sigma Aldrich). OTS was dissolved in a solution of toluene (Sigma Aldrich) at a concentration of 100 µM, then this solution was fluxed inside the channel for 20 min. The improvement of thisfunctionalization treatment in terms of water contact angle is on average up to 101 ± 1°.

**Detection of the four Instants**

The droplet detection by a waveguide is made by a change in light transmission across the channel. Due to diffraction of the light between the continuous and dispersed phases (see Fig. 5), each drop and raise of the signal is characterized by a peak. This peak is used as the identification of the four time instants (t_11,_ t_12,_ t_21,_ t_22_). Notably, this peak does not depend on the geometrical features of the droplets, as shown in Fig. 6 (the label refers the continuous flow rates) the only change with faster and smaller droplets (higher continuous flowrates) is the broadening of the peak, but not the intensity value. Therefore, the instants can be measured in the same way for each velocity and length droplet, and the uncertainty depends only on the acquisition frequency of the DAQ, which is 50KHz for our measurements.


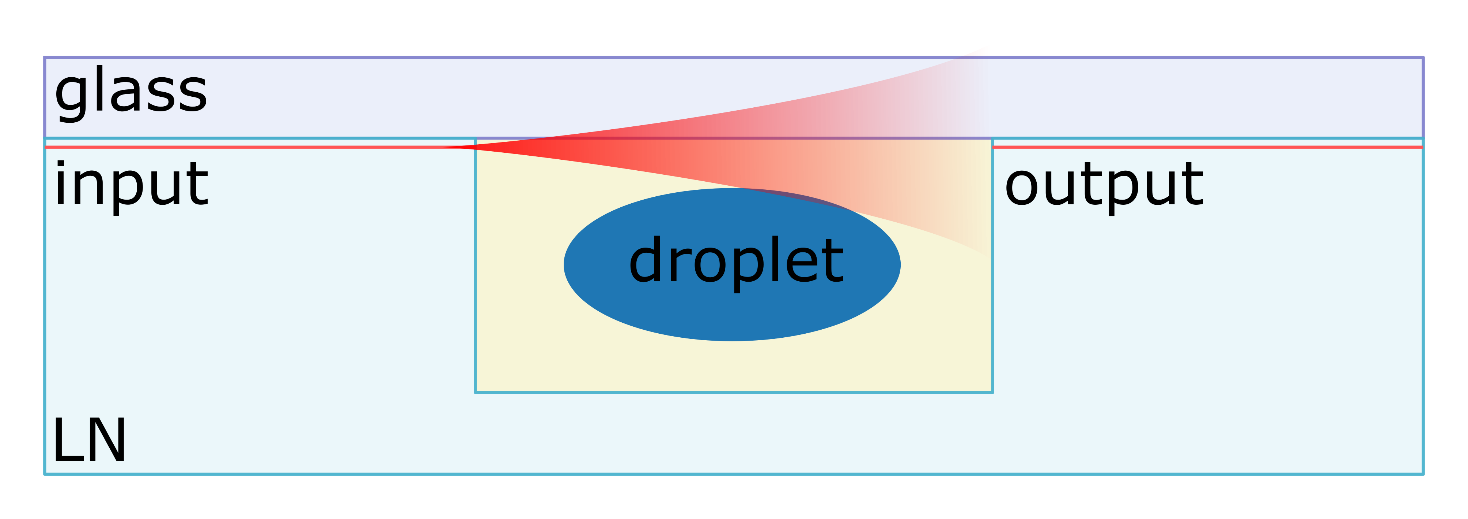


**Figure 5 2D Sketch of the droplet interaction with upper edge located waveguides.** The optical transmission across the channel is modified by the droplet, only at the time, when its section (in the lateral view here considered) is large enough to interact with the light travelling across the channel.


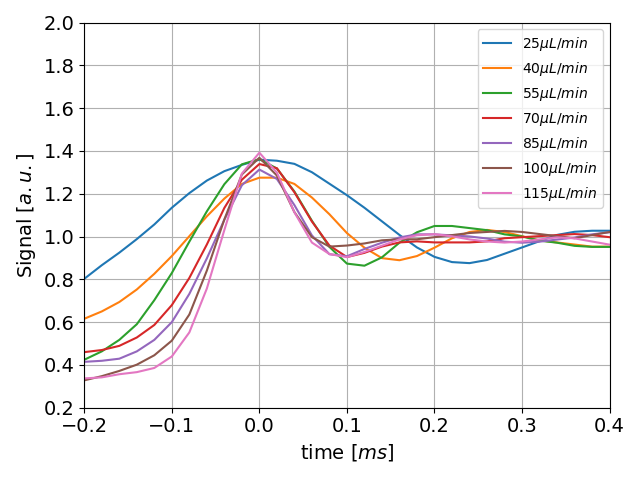


**Figure 6 Determination of the four drop and rise of the signal by the peak.** Seven different rise of the t_22_ are aligned in time, each from droplets produced with Qd = 10 μL/min and Qc in the label. The signal is normalized on the signal of after the droplet flow (i.e. continuous phase in front of both branches of the MZI).

**Single Droplets Detection:**

The error related to the single droplet detection depends on the uncertainty on the identification of the trigger instants. As aforementioned, this identification is univocal for all range tested. For this reason, the uncertainty can be computed by the acquisition frequency as: σ_t_ [ms]=1/√(12) sample rate[KHz], therefore the propagation on the interval result σ_Δt_^2^=2 σ_t_^2^ , and thus the uncertainty on the single droplet velocity is:

σ_v_^2^ = (2W/ Δt^2^) ^2^ σ_Δt_^2^ + σ_2W_^2^ / Δt^2^

Fig. 7 reports the single detection uncertainty comparison between imaging system and MZI, showing by the gray lines how the uncertainty can be improved with higher acquisition frequency.


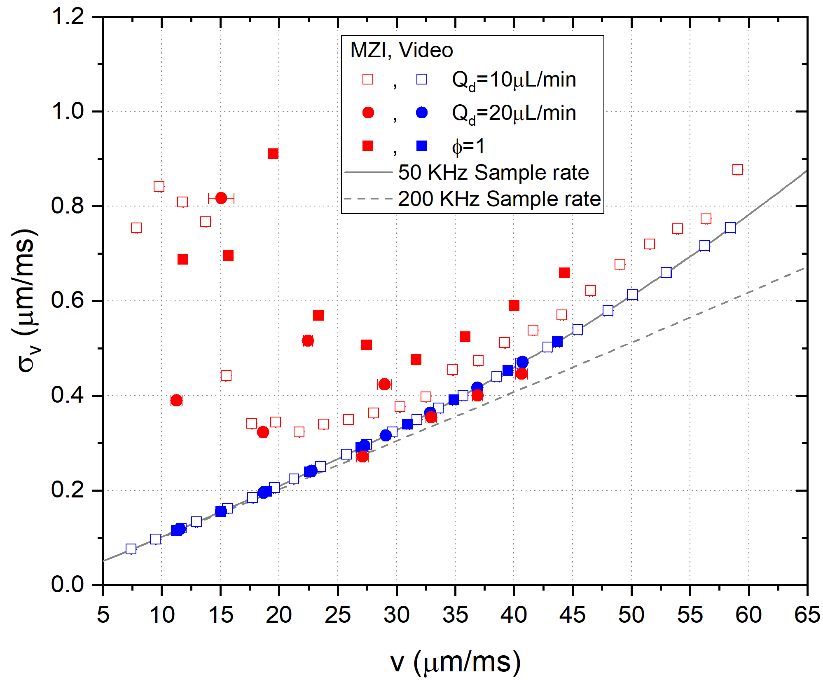


**Figure 7 Graph of the error relative to the velocity estimation of a single object.** The red points refer to the video analysis, where the error values have been extracted as the standard deviation over all the pixel used for the estimation of the velocity of one droplet and over all frames, in which the droplet image is captured. The blue points represent the error on the estimation of the velocity from the optical signal of the MZI-base chip. The two gray lines represent the theoretical calculation of the single error measurements based on the error propagation from equation (1), with sample rate of 50 KHz (full line) and 200KHz (dashed line).

**Length detection of the droplet:**

Since the MZI is located on the upper edge of the channel, only a limited depth of the channel is illuminated by the light from the waveguide. The depth can be calculated with similar consideration aforementioned for the design, in particular for 200 μm wide channels the illuminated depth is 9.6 μm (see sketch in Fig 5). The latter is the maximum distance from the channel top surface for a droplet to be detected. Nevertheless, this limit depends only on the position of the waveguides, which can be change be futher fabrication steps.

This droplet interaction starts only when the section of the droplet is large enough to interact with the lihgt from the waveguide. This feature does not affect the estimator of the trigger nor the velocity estimation, whereas the length measures should consider the addition of the menisci part, which does not interact with the waveguide light. The experimental value is obtained by the intercept of the linear regression of the data between the imaging analysis and the MZI results in Fig. 8 and represents a calibration value (see Fig.8).


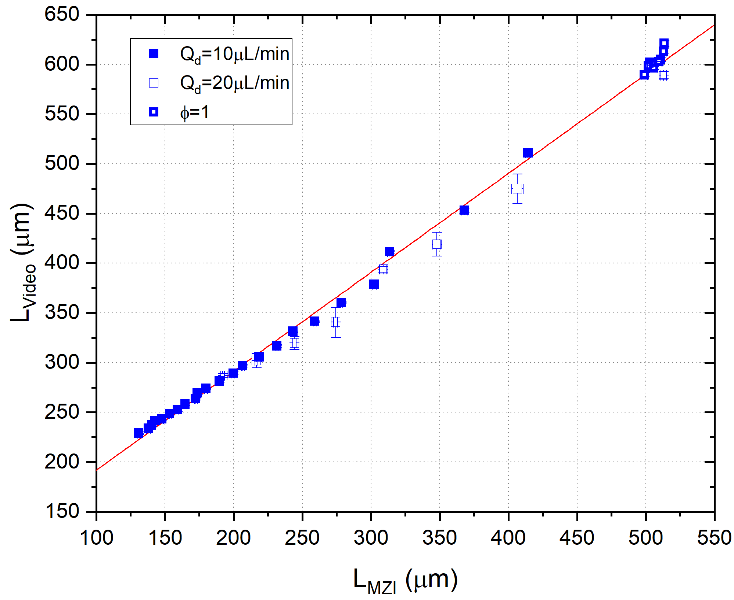


**Figure 8: Comparison on the length detection between the two data acquisition systems (the MZI and the standard imaging system).** All the three datasets presented in the paper are plotted similarly for Fig. 7 in the paper, but for the length of emulsions, the linear interpolation (red line) shows an intercept and slope of 0.996±0.008 and 92±2 µm, respectively.

**References:**

1. Baets, R. & Lagasse, P. E. Calculation of radiation loss in integrated-optic tapers and Y-junctions. *Appl. Opt.* (1982) doi:10.1364/ao.21.001972.
